# Seismic Behavior of Stone Masonry Joints with ECC as a Filling Material

**DOI:** 10.3390/ma14216671

**Published:** 2021-11-05

**Authors:** Wei Hou, Xinghua Dai, Zheyu Yang, Hanhuang Huang, Xiaoli Wang, Pandeng Zheng, Yixin Zhang, Zixiong Guo

**Affiliations:** 1 LETS Holding Group Co., Ltd., Xiamen 361004, China; dzq000000@163.com (X.D.); lily_jessica_ong@sina.com (X.W.); 2 College of Civil Engineering, Huaqiao University, Xiamen 361021, China; zy1102459496@163.com (Z.Y.); HanhuangHuang1209@163.com (H.H.); zpd2102@163.com (P.Z.); zhangyixin@live.xauat.edu.cn (Y.Z.); guozxcy@hqu.edu.cn (Z.G.); 3 Xiamen Institute of Building Research Co., Ltd., Xiamen 361004, China

**Keywords:** stone masonry, joints, engineered cementitious composite (ECC), seismic performance, shear strength model

## Abstract

This paper investigates the seismic behavior of novel stone masonry joints using ductile engineered cementitious composite (ECC) as a substitute for ordinary mortar. Ten stone masonry joints with different types of mortar/ECC were tested under axial and cyclic loads. The filling materials of mortar joints tested included ordinary mortar, polymer mortar, ECC, and composite mortar with two combination proportions of ECC and ordinary mortar. The test results indicated that ECC specimens exhibited a more stable hysteretic response as well as an improvement in strength, deformation, energy dissipation, and strength degradation. The ECC mortar joints maintained integrity during the entire loading process due to the “self-confinement” effect of ECC. A partial substitution of mortar with ECC could provide effective reinforcement and confinement to prevent mortar failure and peeling, thereby allowing such specimens to approach the seismic performance of ECC specimens. Based on the trend of shear strength variations, a corresponding failure process is defined for ECC/mortar joints under cyclic and axial compressive loads, including four distinct stages: linear elastic, crack-developing stage, interface debonding, and friction sliding. New equations are proposed for predicting the shear strength and residual shear strength of the ECC/mortar joints on the basis of the test results, which are validated in the composite mortar specimens.

## 1. Introduction

**Figure 1:**
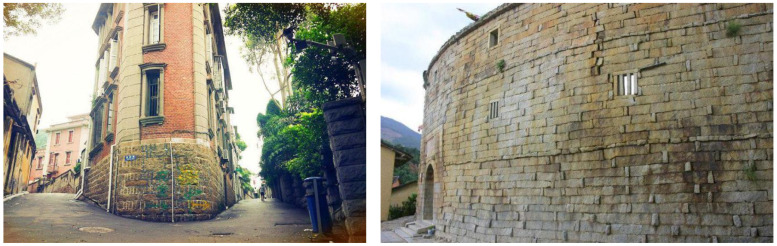
Stone masonry structure in an actual building.

Stone structures are widely used in historical and residential buildings around the world (Figure 1), but their seismic performance has received limited attention. Different from other traditional masonry structures, the strength of the stone is far higher than that of the filling material in mortar joints. By combining experimental and analytical approaches, as well as field investigations of earthquake damage to stone structures, it has been found that the mortar joint of a stone wall is the weakest zone under earthquake [1,2]. Despite past literature having developed different strengthening options of stone masonry joints [3,4,5,6,7,8], the seismic behavior has not yet been assessed. Due to the diversity of masonry technology, the complexity of mortar joint composition, and the difference in material properties, the seismic behavior of stone mortar joints is more complex than that of common masonry mortar joints [2,9]. Therefore, understanding the seismic behavior of stone masonry is of great significance to further protective repair and seismic damage assessments of stone structures.

In order to study the seismic performance of stone mortar joints, the shear behavior and failure mechanism need to be clarified. Earlier experimental studies have been carried out on the bond shear strength and shear failure mode of unit–mortar interfaces [10,11], which indicated that the peak and residual shear strengths are well represented by the Mohr–Coulomb criterion. It is worth noting that the Mohr–Coulomb model is not suitable for irregular masonry; thus, the Mann and Müller criterion [12] for shear failure based on several criteria including the Mohr–Coulomb model will also be affected by the regularity level of stone units. For this type of masonry for determining the shear resistance, the model for diagonal failure by Turnšek and Čačovič [13] based on a phenomenological approach will be considered. Again, Milosevic et al. [14] emphasized the effect of dilatancy deformation in rubble stone masonry, which is caused by the change of the irregularity of the crack surfaces after the masonry joints crack and slide. This effect still exists in regular stone mortar joints [15]. In addition, Angiolilli and Gregori [16] carried out experimental tests as well as numerical investigation to evaluate the shear mechanical parameters of the rubble stone masonry. They found that the confinement pressure and boundary conditions can strongly influence the mechanical response of the test masonry. 

The shear performance of stone mortar joints has been assessed under monotonic loading as well as axial compressive stress for different masonry methods, block types, mortar types, and mortar strengths [15,17]. Vasconcelos et al. [15] conducted a single shear test of mortar masonry stone wall joints under different axial loads. They found a clear influence of the axial compressive stress level on the failure mode and shear strength of the mortar joint. Similar conclusions were proposed by Guo et al. [17]. The results according to a double shear test of the mortar joints of machine-cut stone walls showed the influence of interface treatment modes (between stone and mortar joint); the compressive stress and mortar strength on the shear performance of the mortar joint decrease in turn. 

However, there are few further studies of the seismic behavior of stone masonry joints. Wang et al. [18] conducted an experimental study to evaluate the seismic performance of stone masonry joints under cyclic lateral loads. The test parameters included normal stress and strength and type of mortar. The experimental results were evaluated in terms of the specimens’ hysteretic behavior, deformation characteristics, and shear failure mechanisms. The results showed that the shear behavior of the joints involved four stages: namely, the elastic stage, crack propagation stage, shear strength degradation stage, and frictional sliding stage. The vertical compression deformation increased with the increase in the vertical compressive stress, the amplitude of horizontal displacement, and the number of reciprocating cycles. 

The traditional stone mortar joint consists mainly of laterite mortar and ordinary mortar, and polymer mortar with high strength is often adopted in reinforcement. However, these mortars exhibit a brittle shear failure mode with low shear strength and ductility under lateral force. Engineered cementitious composite (ECC), an ultra-high-performance cement-based composite material, exhibits excellent tensile ductility and crack control ability and has emerged as a promising alternative to mortar in joints. Extensive research has been carried out on the ECC structural members, such as columns [19,20], beams [21,22], and coupling beams [23]. In addition, research [24] on sprayable ECC has proved its potential for structural restoration. These experiments have demonstrated the excellent performance of ECC members, including their high damage tolerance and energy dissipation capacity as well as their excellent ductility. Therefore, applying ECC as a substitute for ordinary mortar in mortar joints would potentially improve both the shear strength and overall ductility of the stone structure under earthquakes. 

Against this background, this study employs an innovative strain-hardening ECC composite using polyvinyl alcohol (PVA) fibers, and fly ash was chosen instead of conventional mortar. The applicability of ECC in elevating the seismic performance of stone masonry joints under lateral loads is not entirely clear. Hence, a detailed experimental and analytical investigation of ECC masonry joints was conducted to determine the suitability of ECC and appraise the influence of axial compressive stress on the seismic behavior of ECC masonry joints. Ten masonry joint specimens consisting of different types of mortar and different levels of compressive stress were tested under increasing lateral cyclic loading in the experiment. The seismic behavior of the test specimens, in terms of hysteretic response, strength, stiffness, and failure mode, was evaluated. Subsequently, a detailed analysis on the failure mechanism of the ECC and masonry joints was conducted. Then, several new equations for shear strength and residual shear strength were proposed based on the test results, which were the experimental fundament of further numerical study.

## 2. Experimental Program

### 2.1. Test Specimens

**Figure 2:**
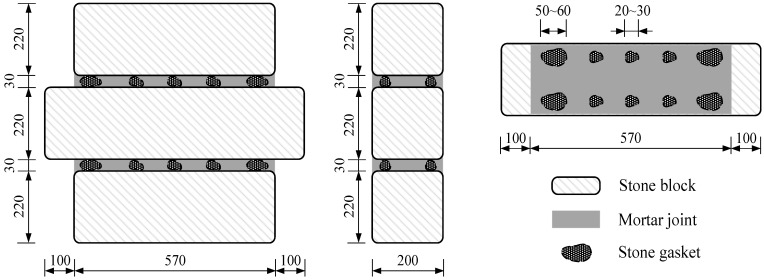
Detailed size of the specimens (unit: mm).

The experimental study examined the seismic behavior of stone masonry joints with different types of mortar under axial compressive stress. The mortar types included ordinary mortar (M), polymer mortar (PM), ECC, and composite mortar with ECC and ordinary mortar (CM). To evaluate the effectiveness of ECC in improving the seismic performance of stone masonry joints, a total of 10 specimens of the same size were constructed and tested under displacement-controlled cyclic loading. The test specimen consisted of three main stone blocks (200 mm × 220 mm) with different lengths (570 mm for upper and lower blocks and 770 mm for the middle one), two mortar joints (30 mm in thickness for each) between the adjacent stones, and ten stone gaskets inside each mortar joint. The detailed size of the specimen shown in Figure 2.

**Figure 3:**
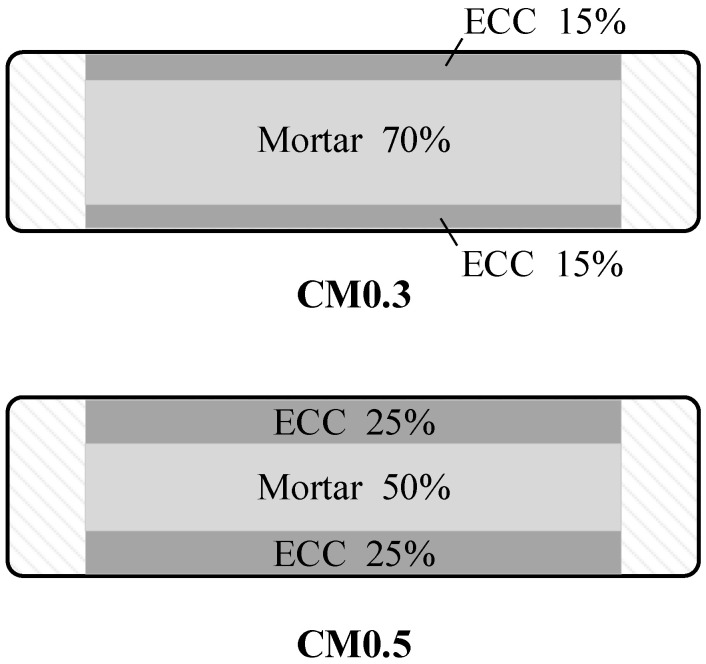
Details of composite mortar.

As seen in Table 1, the test variables included axial compressive stress (0.2 MPa and 0.4 MPa) and mortar types. Three single mortars (M, PM, and ECC) and two different proportions of composite mortars were considered in the experiment (CM0.3 and CM0.5, see Figure 3). For CM specimens, the mortar was partly replaced by ECC on both sides of mortar joints. ECC was conducted as a kind of reinforcement and confinement on the inner mortar to protect the mortar from falling off, resulting in better overall performance. The specimens are identified as follows:

(a) “M”, “PM”, “ECC”, and “CM” represent the mortar type: Mortar, Polymer Mortar, ECC, and Composite Mortar of ECC and mortar, respectively;
(b) Number “0.3” or “0.5” if the specimen consists of ECC and ordinary mortar with the proportions of ECC as 0.3 or 0.5, respectively;
(c) Number “0.2” and “0.4” to represent the axial compressive stress of 0.2 MPa and 0.4 MPa, respectively.

**Figure 4:**
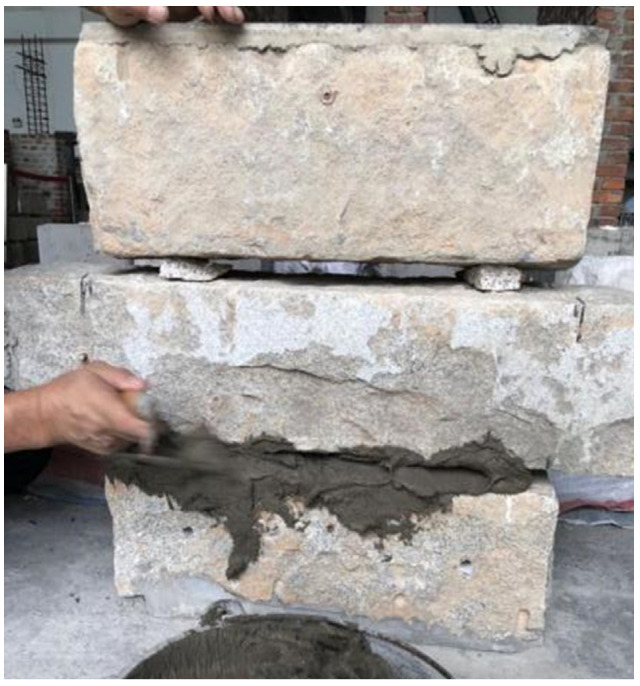
Filling the joints with mortar.

The stone blocks (kind of coarse granite material, collected from the historical unreinforced buildings of stone structure) were stacked with the support of stone gaskets before filling in with the mortar (Figure 4). The mortar was mixed and poured in the laboratory; then, it was filled evenly into the gap between the stone blocks. We pressed properly to ensure the density of mortar. After the completion of the mortar joint masonry, water spray curing was conducted on the surface of the mortar joint for 7 days, following a natural curing time of 28 days before the experiment. In addition, the upper and lower ends of the specimen are leveled with concrete for installation.

### 2.2. Material Properties

The cementitious composition of ECC consisted of P.O. 42.5 grade cement, fly ash, fine sand, silica fume, water reducer, and water (see Table 2). Polyvinyl alcohol (PVA) fibers (with a diameter of 0.038 mm, a length of 12 mm, a tensile strength of 1600 MPa, and a density of 1300 kg/m^3^) were used as reinforcement to the cementitious matrix. The fiber volumetric ratio was 2%. Polymer mortar is kind of a cementitious mortar composed of cement, sands, and dispersed emulsion powder polymers (e.g., acrylate, polyvinyl alcohol, styrene acrylate) to improve the mechanical properties.

**Figure 5:**
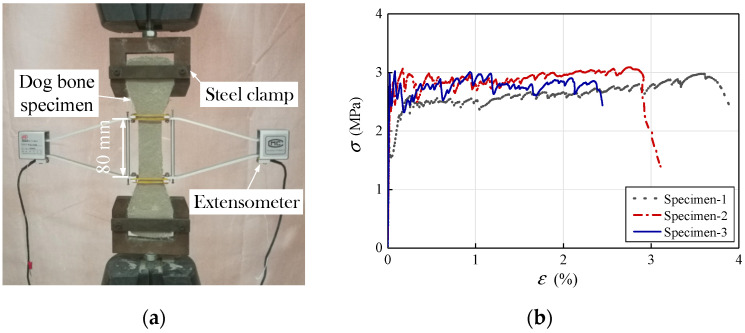
(**a**) Tensile tests on ECC dog bone-shaped specimens; (**b**) Tensile stress–strain curves of ECC specimens.

The compressive strength was tested using four 100 mm cubes following the relevant Chinese standard JGJ/T70-2009 [25]. The average cubic compressive strength of the M, PM, and ECC at the time of column testing was 2.1 MPa, 34.8 MPa, and 22.8 MPa, respectively. “Dog bone”-shaped tensile specimens with an 80 mm long testing region were loaded at a rate of 0.2 mm/min to obtain the tensile strength (Figure 5a), following the relevant Chinese standard JC/T 2461-2018 [26]. The tensile stress–strain curves of ECC shown in Figure 5b) exhibit pseudo strain-hardening behavior. The rupture strain was above 2.5%, demonstrating an excellent tensile ductility. The average tensile strength of ECC was 2.7 MPa.

### 2.3. Test Set-Up and Instrumentation

**Figure 6:**
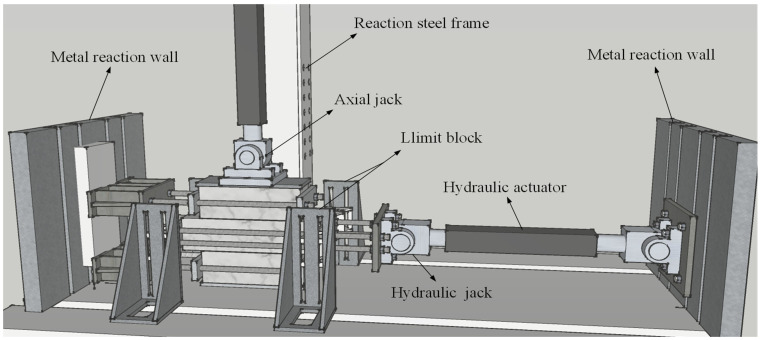
Details of testing rig.

The specimens were tested under displacement-controlled cyclic loading using a 500 kN servo-controlled hydraulic actuator with a stroke length of ±100 mm, as shown in Figure 6. The upper and lower stone blocks were fixed on a metal reaction wall through four screws with steel plates on each side, avoiding moving with the horizontal load. Four limit blocks with a roller were applied to limit the anti-plane displacement of specimens.

**Figure 7:**
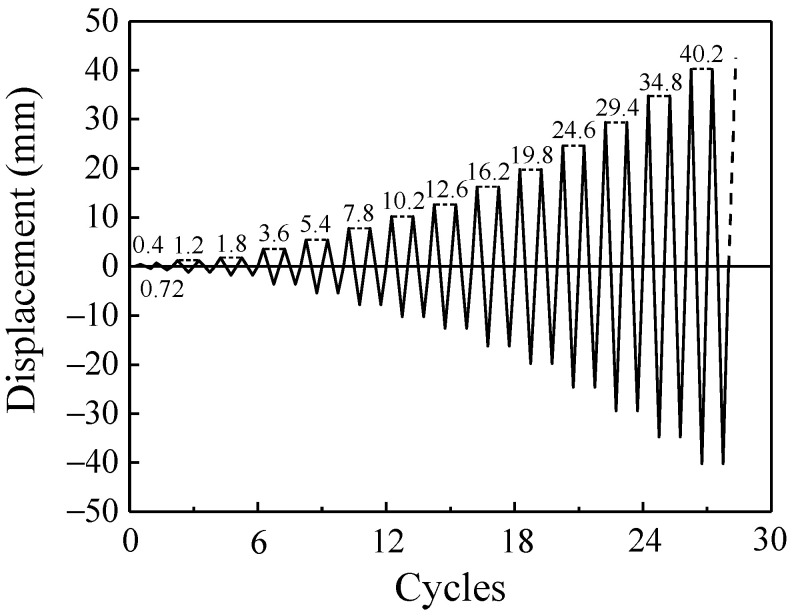
Loading protocol.

The specimens were loaded in two stages: elastic stage and plastic stage following the recommendations of JGJ/T101 [27] and ISO 16670 [28], until the mortar joint attained significant damage or failure (the vertical displacement of the specimen exceeds 40% of the mortar joint’s thickness). Each load cycle was applied only once in the elastic stage of the specimen. Two repeated cycles for each displacement level were applied in the second stage until the specimens were severely damaged. The specific loading protocol is shown in Figure 7.

**Figure 8:**
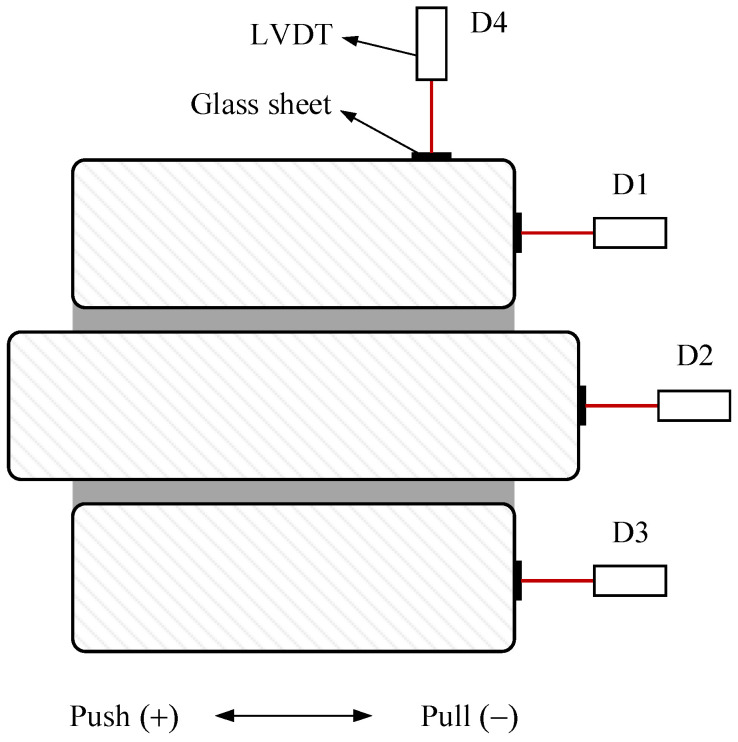
Locations of LVDTs.

The axial load was applied on the top of the specimens, which was kept constant during the entire test. Specimens were instrumented with linear variable differential transducers (LVDTs) and load cells in the actuator arm. As seen in Figure 8, the lateral deformations of the specimens were recorded using LVDTs at the centerline of stone blocks (D1, D2, and D3). One vertical LVDT (D4) was installed on the top of the upper stone to measure the vertical deformation of the specimen.

## 3. Experimental Results and Discussions

### 3.1. Experimental Observations

**Figure 9:**
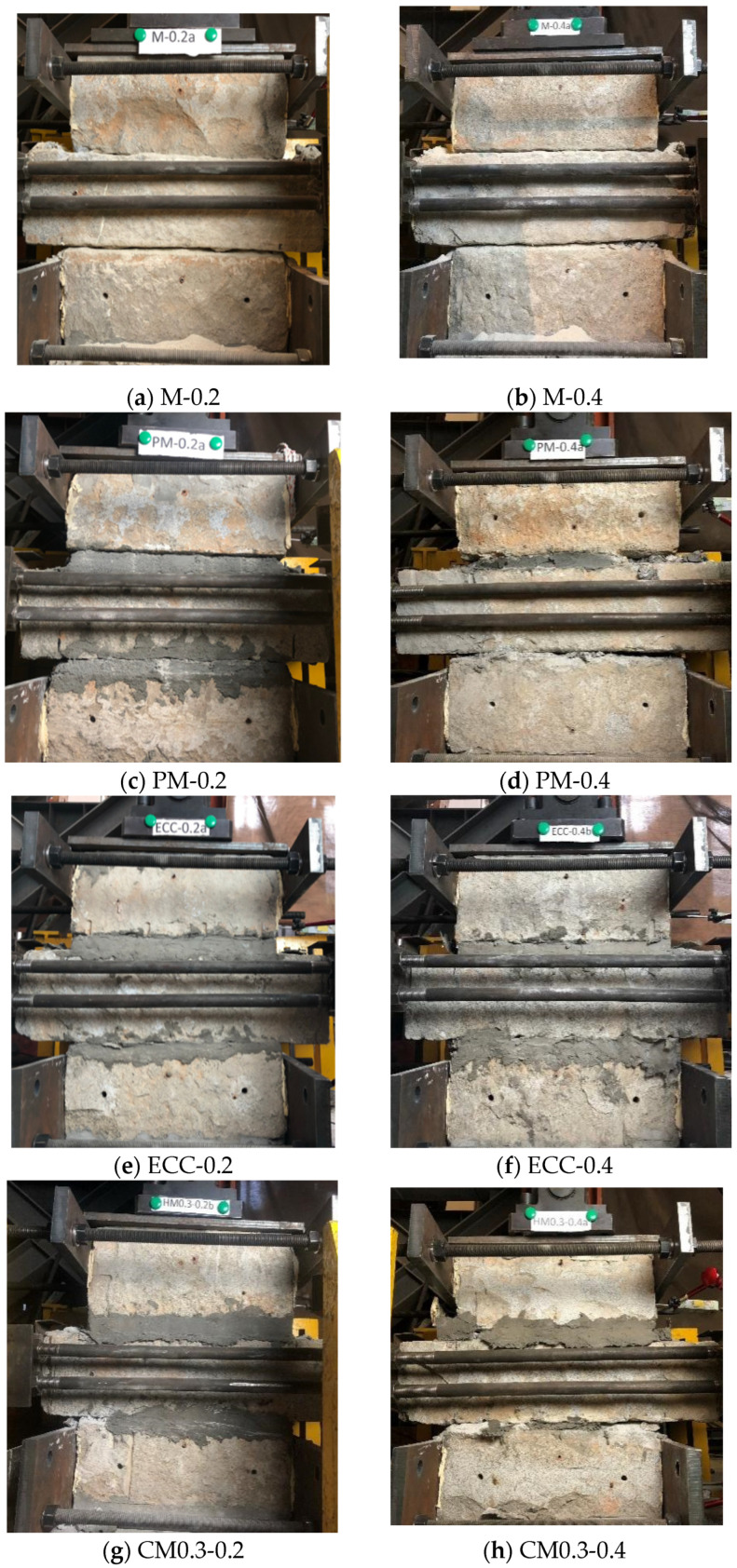

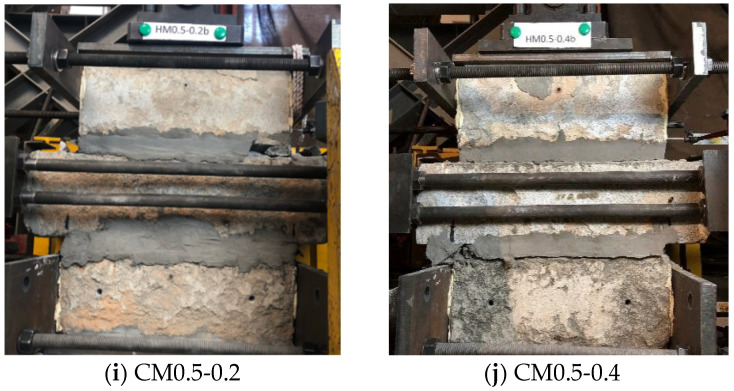
Final damage state of specimens after the test: (**a**) M-0.2, (**b**) M-0.4, (**c**) PM-0.2, (**d**) PM-0.4, (**e**) ECC-0.2, (**f**) ECC-0.4, (**g**) CM0.3-0.2, (**h**) CM0.3-0.4, (**i**) CM0.5-0.2 and (**j**) CM0.5-0.4.

The experimental observations of the specimens were discussed based on the types of mortar (M, PM, ECC, CM0.5, CM0.3) considering the difference of the initiation of cracks followed by their proliferation and final failure mechanisms. Figure 9 shows the damaged state of specimens. For the case of the specimens M-0.2 and M-0.4 under the axial compressive stress of 0.2 MPa and 0.4 MPa, the mortar and stone gasket of the mortar joint have been evidently crushed and extruded. As for PM specimens, a similar failure appearance can be observed under the axial compressive stress of 0.4 MPa, while a relatively intact mortar joint without obvious axial deformation was available to specimen PM-0.2. All the specimens with ECC (ECC, CM0.3, and CM 0.5) maintained the integrity of the mortar joint after the termination of the test, only with a small amount of ECC extruded at both ends of the mortar joint.

**Figure 10:**
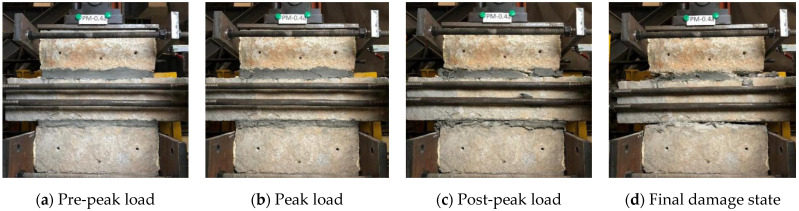
Typical failure process of M/PM specimens (PM-0.4): (**a**) Pre-peak load, (**b**) Peak load, (**c**) Post-peak load and (**d**) Final damage state.

Considering the similarity of the initiation of cracks followed by their proliferation and final failure mechanisms, all specimens were divided into three groups according to the type of mortar. For the case of specimens with ordinary mortar (M and PM), as seen in Figure 10a, the reduction of initial elastic modulus happened at a displacement level of 1.8 mm, when the interface failure of the mortar joint has already occurred. Cracks were initiated as inclined cracks about 45 degrees appeared on the surface of the mortar joint at a displacement level of 3.6 mm, and the lateral load reached the peak load at the same time (Figure 10b). As the displacement level increased (3.6–7.8 mm), the inclined cracks on the surface of the mortar joint gradually developed into inclined network cracks as the lateral load decreased significantly (Figure 10c). At a displacement level of 3.6 mm, the mortar and stone gaskets in the mortar joint were crushed gradually, along with the lateral load tending toward stability. The test was terminated after 19.8 mm displacement when large-scale crushing and extrusion of mortar joint was observed (Figure 10d).

**Figure 11:**
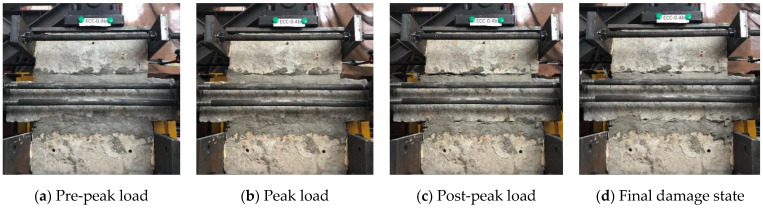
Typical failure process of ECC specimens (ECC-0.4): (**a**) Pre-peak load, (**b**) Peak load, (**c**) Post-peak load and (**d**) Final damage state.

In the specimens with ECC mortar joints (Figure 11), the reduction of initial elastic modulus happened at a similar displacement level (1.8 mm) with ordinary mortar specimens. The peak lateral load of the specimens was initiated at 2.8 mm displacement in ECC-0.2, whereas the peak value was reached at the displacement of 5.2 mm in ECC-0.4. Minor hairline diagonal shear cracks on the surface of the mortar joint were initiated slightly before the peak load’s corresponding displacement. As the monitored displacement level increased, the width of cracks developed within a small range and maintained the integrity of the mortar joint (Figure 11a). In addition, in ECC specimens, the sound of fracturing fibers was noted during the widening and propagation of cracks. The self-confinement effect of fibers in ECC retained the transmission of tensile stress before forming major cracks, thus limiting the crack width and showing high ultimate tensile strain [29,30]. As a result, the cracks of ECC specimens were dense until the end of the test (Figure 11b–d).

**Figure 12:**
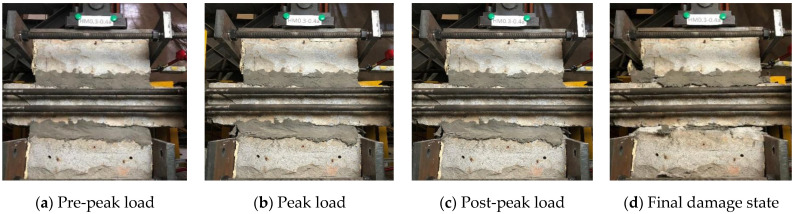
Typical failure process of CM specimens (CM0.3-0.4): (**a**) Pre-peak load, (**b**) Peak load, (**c**) Post-peak load and (**d**) Final damage state.

For the case of composite mortar specimens, as the ECC on both sides of the mortar joint protected the mortar in the middle, these specimens could also show prominent integrity as well as good performance. Early experimental phenomena were similar to that of ECC specimens (Figure 12a) until reaching the peak load (Figure 12b). For the CM0.3 specimens, minor shear cracks were initiated first on the surface of ordinary mortar in the middle, which was followed by ECC being gradually squeezed out due to the expansion of mortar inside (Figure 12c), and then, the tests were terminated (Figure 12d). In CM0.5 specimens, a higher proportion of ECC formed stronger confinement to the mortar in the middle. As a result, the whole composite mortar joint remained complete without ECC peeling off.

### 3.2. Hysteretic Responses

**Figure 13:**
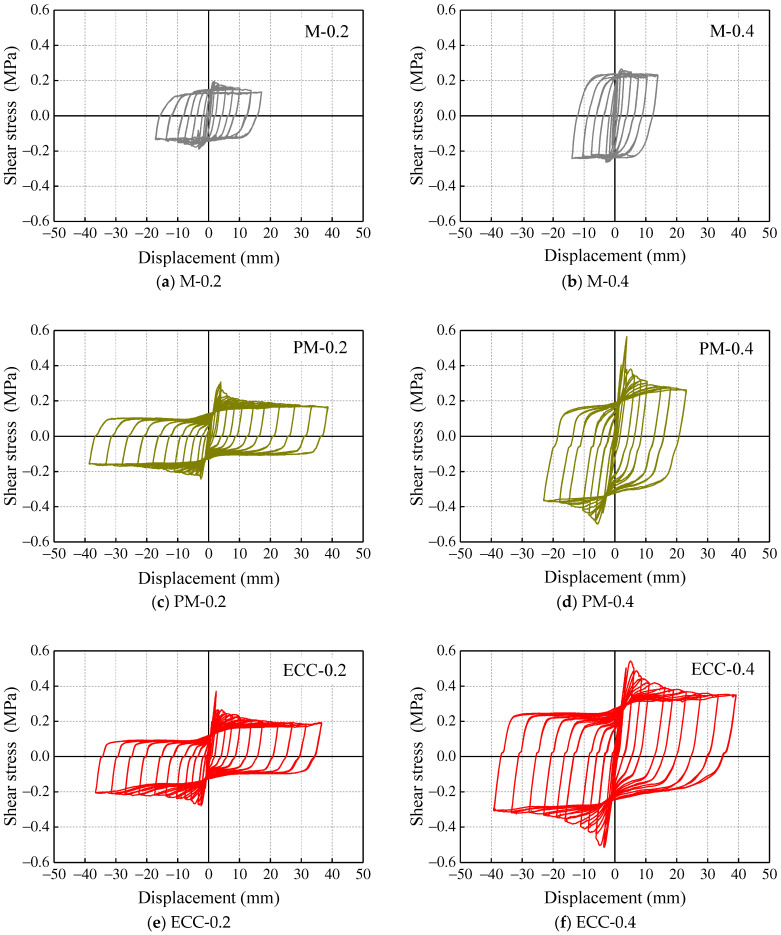

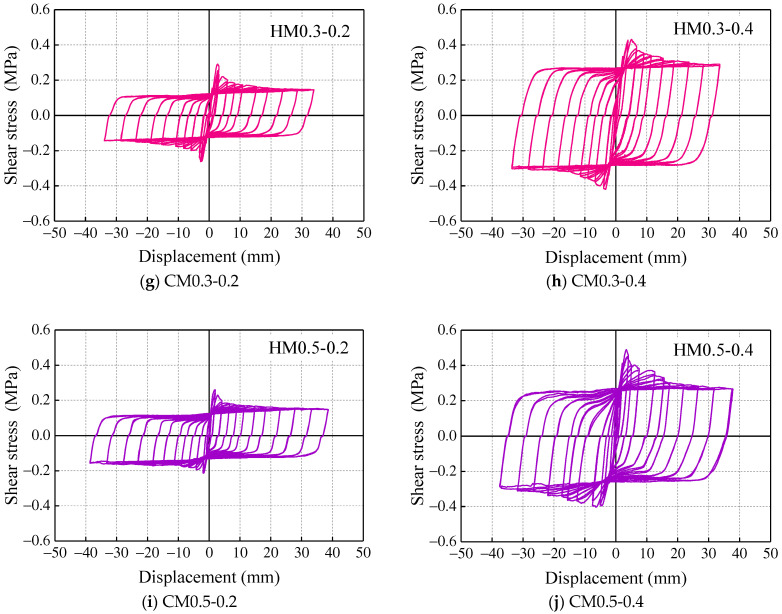
Hysteretic response of specimens: (**a**) M-0.2, (**b**) M-0.4, (**c**) PM-0.2, (**d**) PM-0.4, (**e**) ECC-0.2, (**f**) ECC-0.4, (**g**) CM0.3-0.2, (**h**) CM0.3-0.4, (**i**) CM0.5-0.2 and (**j**) CM0.5-0.4.

The hysteretic response of all the specimens is shown in Figure 13, and the experimental results of the tested specimens are summarized in Table 3. The compressive strength of mortar has hardly affected the shear strength of the specimens, which is mainly contributed by the axial compressive stress (see *τ_m_*/*σ*_n_ in Table 3). Similarly, the ECC did not enhance the lateral load-carrying capacity of the specimens than the M and PM specimens, but the specimens exhibited a much more stable hysteretic response. For the case of M specimens, the spalling of the mortar joint happened quickly and led to a low displacement level (less than 20 mm). In addition, the extremely low compressive strength of mortar (2.1 MPa) limited the potential improvement of the shear strength of the specimen even under a higher axial compressive stress. 

When it comes to PM specimens with higher compressive strength of mortar (34.8 MPa), PM-0.2 exhibited a stable hysteretic response without spalling of the mortar joint. However, a higher axial compressive stress seemed to be detrimental to PM specimens where the brittle failure happened again, and all the mortar joints have peeled off after the test. A similar phenomenon was observed in CM0.3-0.4 due to the low proportion of ECC in composite mortar, which failed to provide adequate protection for inner mortar; a slight pinching appeared in the push direction of the hysteretic response curve. Correspondingly, ECC was extruded in the later stage of loading due to the cracking and swelling of mortar. Considering the integrity of the mortar joint in CM0.3-0.2, it follows that the mortar joint is more likely to fail with the increase in axial pressure. Moreover, in the same axial compressive stress specimens, CM0.5 observed a lesser amount of pinching and, consequently, higher deformation capacity and energy dissipation due to a higher ECC proportion in the composite mortar than CM0.3. In that case, the brittle nature of the conventional normal mortar and ECC ductile behavior was pragmatic from the hysteretic response comparison.

### 3.3. Skeleton Curves

**Figure 14:**
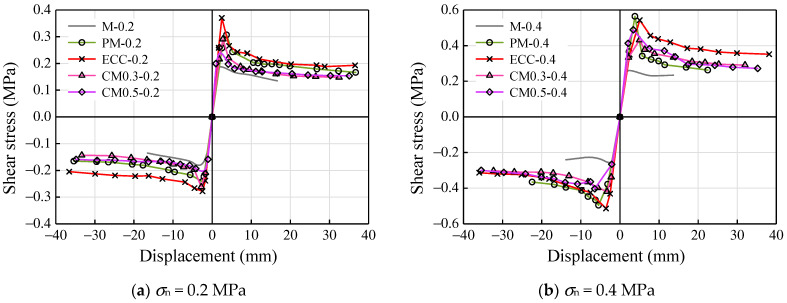
Shear stress–displacement responses: (**a**) *σ*_n_ = 0.2 MPa and (**b**) *σ*_n_ = 0.4 MPa.

Figure 14 shows the comparisons of the shear stress–displacement response of specimens. It was observed that specimens using ECC showed higher deformation ability, and an increase in the axial compressive stress increased the load-carrying capacity of the specimens. In the initial stage of the test, the skeleton curves of all kinds of mortar joints maintained linear growth, with the elastic modulus values very close to each other. ECC specimens saw the highest value of shear strength in all the specimens for 0.2 MPa axial compressive stress. As for high axial compressive stress (0.4 MPa), PM-0.4 and ECC-0.4 had a similar shear strength, while the degradation in strength in the ECC specimen was more gradual than that of the PM specimen (Figure 14b).

The self-confinement effect of fibers maintained the integrity of ECC in the post-peak region, and this effect preserves the transmission of stress through the fibers without any obvious degradation in strength. Compared to CM and M specimens, the partial substitution of ordinary mortar with ECC could increase the shear strength directly. Mortar reinforced by ECC presented the seismic performance gradually close to that of ECC specimens with the increase in mortar replacement ratio. As a further increase in displacement level, the shear strength of specimens tended to be stable, which meant the friction slip stage of loading.

### 3.4. Stiffness Degradation

**Figure 15:**
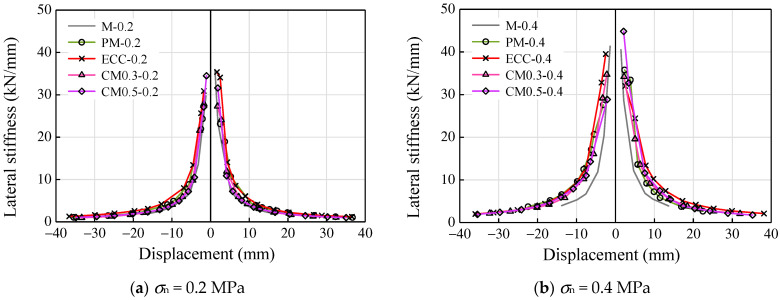
Lateral secant stiffness–displacement responses: (**a**) *σ*_n_ = 0.2 MPa and (**b**) *σ*_n_ = 0.4 MPa.

Figure 15 shows the secant stiffness of the specimens at different displacement levels. The initial lateral stiffness of all the specimens increased with an increase in axial compressive stress. This may be due to the higher axial compressive stress leading to a higher frictional force between the stone blocks and mortar joint, which improves the lateral resistance of specimens. As seen in Figure 15a, all the specimens tended to present adjacent trends as well as similar initial stiffness around 35 kN/mm under the axial compressive stress of 0.2 MPa. For the case of the specimens under 0.4 MPa compressive stress, specimen M-0.4 exhibited a rapid stiffness degradation due to the low compressive strength of mortar. The application of ECC in composite mortar had significantly increased the lateral secant stiffness of specimens, while the degradation in strength was more gradual.

### 3.5. Energy Dissipation and Equivalent Viscous Damping

**Figure 16:**
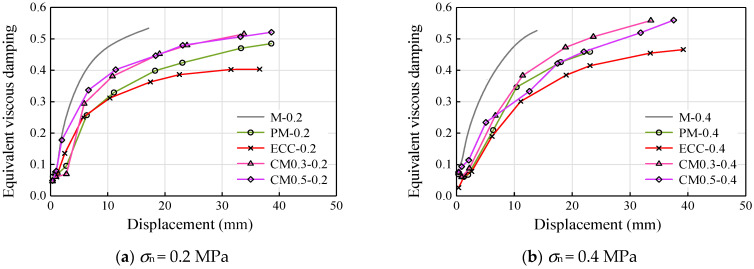
Equivalent viscous damping: (**a**) *σ*_n_ = 0.2 MPa and (**b**) *σ*_n_ = 0.4 MPa.

**Figure 17:**
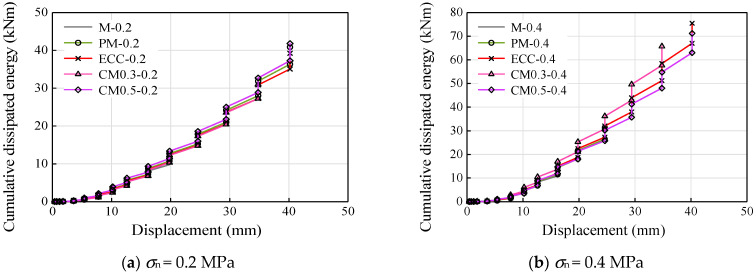
Cumulative energy dissipation: (**a**) *σ*_n_ = 0.2 MPa and (**b**) *σ*_n_ = 0.4 MPa.

Figure 16 and Figure 17 show the variation of equivalent viscous damping and cumulative energy dissipated for different displacement levels, and Table 4 shows the variation of cumulative energy of specimens after the test. The equivalent viscous damping (*β*_eq_) was estimated as per the recommendations of ATC [31] and Hou et al. [32], as given in Equations (1) and (2).

(1) $$K_{\mathrm{eq}}=\frac{V^{+}-V^{-}}{\Delta^{+}-\Delta^{-}}$$

(2) $$\beta_{\mathrm{eq}}=\frac{2}{\pi }\frac{E_{c}}{K_{\mathrm{eq}}{\left(\Delta^{+}-\Delta^{-}\right)}^{2}}$$

where *K*_eq_ is the effective secant stiffness, Δ^+^ and Δ^−^ are the displacements corresponding to maximum forces *V*^+^ and *V*^−^ in pull and push directions, respectively, and *E_c_* represents the energy dissipated per displacement cycle level. The increase in the equivalent viscous damping ratio (Figure 16) was found to be similar with different axial compression stress. All the specimens saw the rapid increase in equivalent viscous damping ratio until a displacement of about 10 mm, where the shear stress of specimens started approaching a relatively stable platform.

As seen in Table 4, there was no significant increase in the total energy dissipated by the M specimens with an increase in the axial compression stress, which was increased by about 19%. It can be explained by the low strength of ordinary mortar, which was destroyed early under high compression stress. As for the decrease in the total dissipated energy of PM specimens, a similar reason was appropriate as the brittle properties of mortar even with high compressive strength. As a result, a much earlier failure of mortar in PM-0.4 (Δ = 23.5 mm) was observed compared to that of PM-0.2 (Δ = 38.9 mm). For the case of ECC and CM specimens, the higher ductility of ECC ensures the integrity of the mortar joint; thus, a steady improvement in the total energy dissipated (approximately 1.7–2.1 times) was observed with an increase in the axial compression stress.

From the results of cumulative energy dissipation for various compression stress (Figure 17), all the specimens with different types of mortars showed a very similar trend with the axial compression stress of 0.2 MPa. As the shear stress was mainly sliding friction after bond failure, the energy dissipation for each loading cycle saw a steady growth on the premise of the mortar joint remaining relatively intact. In this case, low axial compression stress, which was a benefit to the integrity of the mortar joint, led to the result shown in Figure 17a. For the specimens with axial compression stress of 0.4 MPa, only the ECC and CM series showed sustained growth in cumulative energy dissipation as the displacement increase. CM0.3-1.4 showed the fastest growth rate with the price of mortar peeling off. The cumulative energy dissipation of CM0.5-0.4 was quite close to that of the ECC one, in both trend and the total value of cumulative energy dissipation, indicating the effectiveness of partially replacing mortar with ECC by 50%.

## 4. Mechanical Behavior and Shear Strength of Mortar Joint

### 4.1. Failure Process

**Figure 18:**
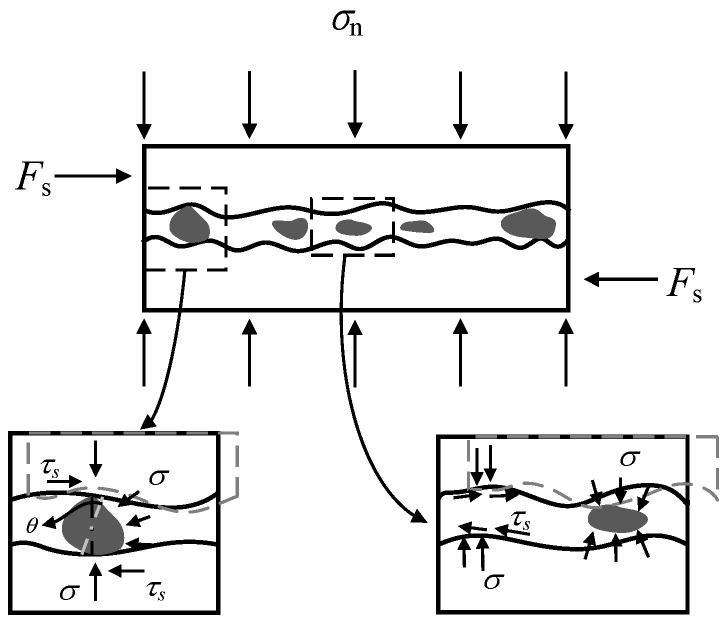
Failure mechanism of mortar joint before peak load (stages I and II).

**Figure 19:**
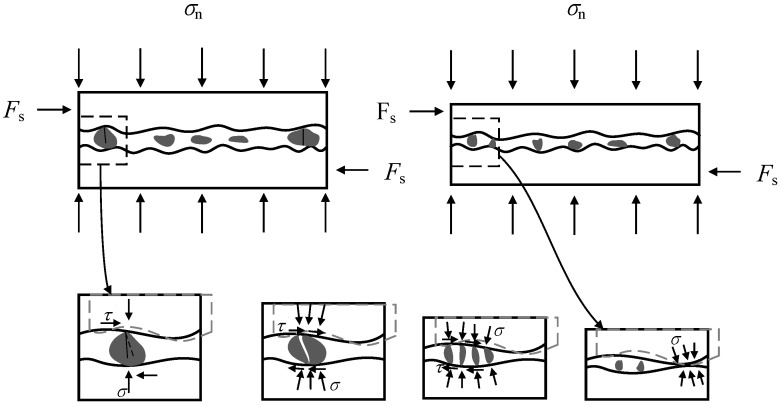
Failure mechanism of mortar joint after peak load (stage III).

**Figure 20:**
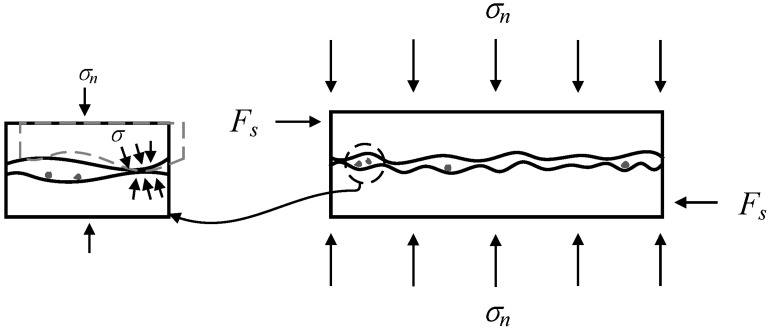
Failure mechanism of mortar joint in friction-sliding stage (stage Ⅳ).

Previous studies suggested that the strain localization caused by splitting cracks in mortar is one of the main reasons leading to the failure of mortar joints. Therefore, based on the mechanical behavior of different types of mortars, a failure process of stone masonry joints under lateral and axial loads was proposed, as shown in Figure 18, Figure 19 and Figure 20. The entire process is divided into four stages: (I) linear elastic stage, (II) crack-developing stage, (III) interface-debonding stage, and (Ⅳ) friction-sliding stage.

(I) Linear elastic stage

For mortar and ECC specimens, before their shear strain reaches the cracking strain of mortar or ECC, some microcracks have already formed at the locations of internal defect in the cement matrix under compression. In this stage, interface bonding maintains integrity among different components (mortar, stone block, and stone gasket), and the damage of materials is negligible. 

(II) Crack-developing stage

As the lateral displacement increases, the principal tensile strain of cement-based materials increases. Under the combined action of axial pressure and lateral load, oblique tension cracks along 45 degrees emerge in the mortar joints. Due to the higher ultimate tensile strain of ECC, diagonal cracks appear later than that of ordinary mortar, which is usually around the peak load. In addition, the interface bonding force between the mortar joint and stone blocks gradually reached the maximum as the displacement increases, while the horizontal interface cracks occur locally at the same time. This effect leads to a lower lateral stiffness when approaching the peak load compared to the initial value. Considering that the axial compressive stress is the main contributor to the interface bonding force, the decrease in the lateral stiffness of the specimens is more prominent under higher compressive stress. 

(III) Interface-debonding stage

After reaching the peak lateral load, the interface-bonding behavior is gradually replaced by the friction-sliding behavior between the mortar–stone interface, which is accompanied by the development of horizontal interface cracks. In addition, the diagonal cracks in mortar joints develop rapidly under large displacement, especially in the ordinary mortar joint with low compressive strength or under high compressive stress. For specimens M-0.2, M-0.4, PM-0.4, and CM0.3-0.4, the cumulative damage due to the crushing of the mortar and stone gasket leads to a rapid decrease in shear strength. As seen in Figure 19, the mortar and stone gasket are broken and squeezed out of the mortar joint during this stage, along with the increase in axial deformation of the whole specimen. In the case of other specimens, the mortar joints maintain relative integrity without spalling. When the interface between stone block and mortar joint is completely separated, then the shear stress reaches a stable value, which implies the end of this stage.

(Ⅳ) Friction-sliding stage

In this stage, the interface bond is completely invalid and replaced by friction-sliding behavior. For specimens M-0.2, M-0.4, PM-0.4, and CM0.3-0.4, the mortar in the joint is destroyed and squeezed out, and the stone blocks on both sides of the mortar joint come into contact. Mechanical occlusion as well as friction slip occurred simultaneously in these specimens (Figure 20). For the case of other specimens without obvious mortar spalling, the sliding friction between the stone block and mortar joints is the main component of shear strength of specimens. In other words, the mortar joints have already quitted the work regardless of whether the mortar is peeling or not.

### 4.2. Shear Strength of Mortar Joint

As discussed in the failure process of mortar joint, bond slip behavior on the interface between the mortar joint and the stone block is the main shear mode before the peak load, where the mortar joint remains basically intact. It is generally considered that the shear failure of masonry mortar joint conforms to Mohr–Coulomb theory. The shear strength of mortar joint is given in Equation (3).

(3) $$\tau =c+\sigma_{n}\tan\varphi$$

where *c* is the cohesive force, representing the shear strength when the axial compressive stress *σ*_n_ = 0; *φ* represents the interior friction angle, which is related to the physical property of the interface between mortar joint and stone block. The internal friction coefficient (tan*φ*) can be assumed as a constant (*B*) for each type of mortar. Previous studies have shown that the cohesive force is the function of mortar strength (*f_c_*′); according to Chinese standard GB 50010 [33], the relationship between cohesive force and compressive strength of mortar is assumed in Equation (4).

(4) $$c=A\sqrt{f_{c}^{\prime }}$$

where *A* represents the undetermined coefficient relating to the performance of the mortar. Wang [18] carried out an experimental program consisting of nine stone mortar joint specimens with three different compressive strengths under various axial compression. The size of test specimens and the source of stone blocks in the experiment are the same as those of the present study.

Based on the above, the shear strength of mortar joints can be assumed as shown in Equation (5). The empirical formulas are obtained based on the experimental data of ordinary mortar (M and PM) in the present study as well as Wang’s study, as given in Equation (6), and the shea strength for ECC specimens is given in Equation (7).

(5) $$\tau =A\sqrt{f_{c}^{\prime }}+B\sigma_{n}$$

(6) $$\tau_{M}=0.039\sqrt{f_{c}^{\prime }}+0.778\sigma_{n}$$

(7) $$\tau_{E}=0.023\sqrt{f_{c}^{\prime }}+1.050\sigma_{n}$$

**Figure 21:**
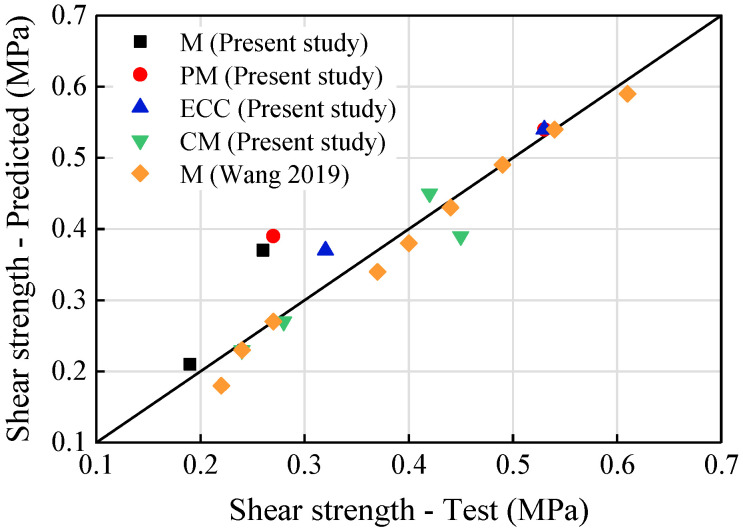
Comparison of the shear strength between the predicted results and the test data.

For the case of composite mortar, a linear combination of Equations (6) and (7) is conducted based on their proportion in composite mortar as given in Equation (8).

(8) $$\tau_{\mathrm{CM}}=m\tau_{M}+n\tau_{E}$$

where *m* and n represent the proportion of the mortar and ECC in the composite, respectively, ranging from 0 to 1. The result of the predicted value according to Equations (6)–(8) are compared with the test data in Table 5 and Figure 21.

As seen in Table 5 and Figure 21, the shear strengths predicted by the proposed equations are in good agreement with the experimental values for most of the specimens. The equations overestimate the values for specimens M-0.4 and PM-0.2 by about 40%. This considerable difference may be due to the early damage caused by the internal defects inside the mortar joints. The predicted strengths of CM series are in good accordance with the measured ones, indicating that the shear stress in composite mortar joint is contributed by both the ECC and the mortar according to their proportions.

### 4.3. Residual Shear Strength of Mortar Joint

When the experiment has progressed to stage Ⅳ (friction-sliding stage), the shear stress on the interface between the mortar and block has gradually decreased to a relatively stable value. The cohesive force is supposed to disappear at this stage, and the shear stress is mainly represented by the sliding friction on the interfaces. In this case, Equations (6)–(8) regardless of the value of cohesive force could be adopted to estimate the residual shear strength (*τ*_e,r_) of specimens. Table 6 shows the result of predicted values compared with test data, and the calculation results overall are in good agreement with the experiment. It is also worth noting that the predicted values tend to slightly overestimate the residual shear strength for ECC specimens. One possibility is the change of internal friction angle before and after the experiment.

Generally, the interior friction angle is mainly determined by the relative relationship between the axial compressive stress and the shear stress. When the roughness of friction slip interface cannot be ignored, the influence of the roughness on the friction slip interface should be considered. In this experiment, the interface of mortar joint is uneven, and the geometric friction angle (*ϕ*) of the interface is considered. In consideration of the same batch of stone blocks as that in Wang’s [18] study, the same value of geometric friction angle (*ϕ*) is taken as 7.9 degrees [34] based on the average value of concave–convex on the surface of six test stone blocks (7.9 mm). In addition, the physical friction angle (*θ*) is proposed to indicate the relationship between the axial compressive stress and the shear stress [34,35,36]. The relationship between the interior friction angle (*φ*), geometric friction angle (*ϕ*), and physical friction angle (*θ*) is given as: tanφ=tan(ϕ+θ).

**Figure 22:**
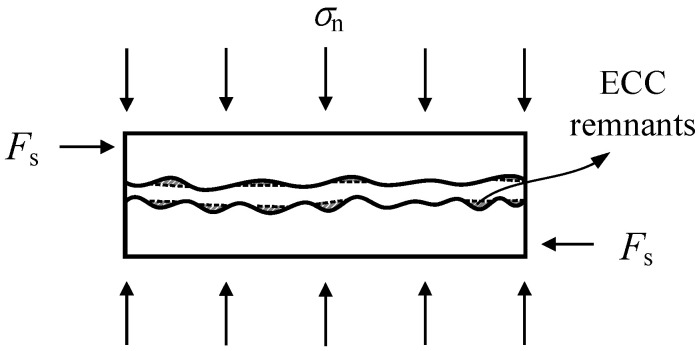
ECC remnants in the indentation of stone surface during stage Ⅳ.

Considering the potential change of surface roughness degree during the loading process, especially in stage Ⅳ, the geometric friction angle (*ϕ*) may have changed at the same time. For the case of ECC specimens, after the formation of horizontal joint cracks, the peeled ECC in the ECC–stone block interface remains bonded to the stone and fills the indentation of it. As a result, the regularity level of stone blocks tend to decrease to some degree (Figure 22). When assuming the geometric friction angle drops from 7.9 degrees to 0, then the internal friction coefficients for mortar and ECC are 0.578 and 0.795, respectively. The residual shear strengths considering geometric friction angle as 0 (*τ*_p,r0_) are calculated in Table 6. Owing to the mechanical occlusion behavior in ordinary mortar joint during stage Ⅳ (see Figure 20), the geometric friction angle in these specimens sees a higher value than that of ECC specimens. Therefore, combined with the results in Table 6, direct calculation by Equation (6) with deduction of cohesive force is suggested, while a reduction factor of 0.83 (the reciprocal of the average of *τ*_p,r_/*τ*_e,r_) for ECC ones is proposed when predicting the residual shear strength of mortar joints.

## 5. Conclusions

The suitability of ECC concrete as an alternative to mortar as a filling material to improve the seismic response of stone mortar joints was assessed by conducting a comprehensive experimental and analytical investigation of different types of mortar and different levels of axial compressive stress. The following conclusions can be drawn based on the results of this study:

- The ECC specimens exhibited a more stable hysteretic response than the M and PM specimens, while the contribution of ECC to the specimens’ load-carrying capacity of specimens was comparable to that of polymer mortar. On the other hand, high toughness and strain-hardening ECC concrete exhibited significantly better deformation and strength degradation characteristics in the post-peak region when compared to the conventional mortar specimen.
- The ECC mortar joint maintained integrity even under high axial compressive stress and large displacement, which leads to higher energy consumption capacity and lower axial deformation. Therefore, the ECC mortar is beneficial to maintain the stability of the overall stone structure under earthquakes.
- The partial substitution of ordinary mortar with ECC can effectively improve the shear strength and strength retention capacity of mortar joints, as the ECC on both sides of the mortar provided reinforcement and confinement to prevent mortar failure and peeling. The CM specimens with a substitution rate of 50% exhibited seismic behavior similar to that of the ECC specimens.
- According to the trend of shear strength variations of mortar joints, a shear failure process of ECC and mortar joint was defined. It includes four distinct stages: (I) linear elastic stage, (II) crack-developing stage, (III) interface-debonding stage, and (Ⅳ) friction-sliding stage. The proposed mechanism accounts for the effects of the different shear behaviors of mortar and ECC and the axial compressive stress on the failure modes.
- Based on the testing data and existing research, a set of new equations was proposed for predicting the shear strength and residual shear strength of ECC/M mortar joints. The compressive strength of mortar and axial compressive stress were taken as the main parameters in the proposed equations. The shear strength of CM mortar joint was contributed linearly by ECC and mortar according to their respective combination proportions.

The limited experimental and analytical investigation ascertained that ECC enhanced the ductile shear performance of the stone mortar joint and afforded a potential reinforcement of and alternative solution to mortar to improve the seismic performance of stone masonry structures. The numerical study will be advanced to developing a shear-compression constitutive model of stone masonry joints.

## Figures and Tables

**Table 1:** Design parameters of the test specimens.

Specimens	Axial Compressive Stress *σ*_n_ (MPa)	Types of Mortar	Cube Strength of Mortar *f*_c_′ (MPa)	Modulus of Mortar
M-0.2	0.2	Mortar	2.1	2.17
M-0.4	0.4			
PM-0.2	0.2	Polymer mortar	34.8	3.45
PM-0.4	0.4			
ECC-0.2	0.2	ECC	22.8	6.67
ECC-0.4	0.4			
CM0.3-0.2	0.2	ECC & Mortar (0.3:0.7)	22.8 & 2.1	6.67 & 2.17
CM0.3-0.4	0.4			
CM0.5-0.2	0.2	ECC & Mortar (0.5:0.5)	22.8 & 2.1	6.67 & 2.17
CM0.5-0.4	0.4			

**Table 2:** Mix proportions of the concrete matrix.

Mortar Types	Cement (kg/m^3^)	Silica Fume (kg/m^3^)	Grade Ⅱ Fly Ash (kg/m^3^)	Quick Lime (kg/m^3^)	Sand (kg/m^3^)	Water (kg/m^3^)	Super Plasticizer (kg/m^3^)
Mortar	80 (P.O 32.5)	–	–	240	1300	270	–
Polymer mortar	490 (P.O 42.5)	–	–	–	1300	185	8
ECC	240 (P.O 42.5)	160	800	–	550	300	6

**Table 3:** Experimental results of the specimens.

Specimen	*τ*_0_ (MPa)	Δ_0_ (mm)	*τ*_m_ (Mpa)	Δ_m_ (mm)	*τ*_0.8m_ (Mpa)	Δ_u_ (mm)	$\tau_{m}/\sqrt{f_{c}^{\prime }}\;(10^{-1})$	*τ*_m_/*σ*_n_	*μ*	Spalling of Mortar Joint
M-0.2	0.17	1.15	0.19	2.55	0.16	6.30	1.32	0.96	5.48	YES
M-0.4	0.25	1.39	0.26	2.52	0.23	7.60	1.82	0.66	5.47	YES
PM-0.2	0.24	2.32	0.27	3.10	0.22	6.85	0.46	1.37	2.95	NO
PM-0.4	0.37	2.73	0.53	4.66	0.42	6.07	0.90	1.33	2.22	YES
ECC-0.2	0.25	1.72	0.32	2.48	0.26	4.30	0.68	1.62	2.50	NO
ECC-0.4	0.39	2.49	0.53	4.32	0.42	9.81	1.11	1.32	3.94	NO
CM0.3-0.2	0.21	1.76	0.28	2.77	0.22	4.40	0.96	1.38	2.50	NO
CM0.3-0.4	0.34	2.22	0.43	4.15	0.35	8.51	1.47	1.06	3.83	YES
CM0.5-0.2	0.18	0.99	0.24	1.83	0.18	5.74	0.67	1.18	5.80	NO
CM0.5-0.4	0.34	2.10	0.45	4.92	0.37	11.11	1.26	1.12	5.29	NO

Note: *τ*_0_ represents the stress when the first crack was initiated corresponding to displacement (Δ_0_); *τ*_m_ represents the maximum shear stress, and Δ_m_ is the corresponding displacement; *τ*_0.8m_ represents the stress corresponding to 80% maximum, and Δ_u_ is the corresponding displacement; *f_c_*′ represents the average cubic compressive strength of mortar; *σ*_n_ represents the axial compressive stress of test specimens; *μ* represents the ductility displacement calculated as Δ_u_/Δ_0_.

**Table 4:** Total dissipated energy of the test specimens.

Specimens	Total Dissipated Energy (kNm)	D-Value
M-0.2	11.40	19%
M-0.4	13.51	
PM-0.2	40.94	−23%
PM-0.4	31.38	
ECC-0.2	39.31	96%
ECC-0.4	77.21	
CM0.3-0.2	31.03	112%
CM0.3-0.4	65.71	
CM0.5-0.2	41.90	70%
CM0.5-0.4	71.43	

**Table 5:** Experimental and predicted results of shear strength.

Research	Specimen	*σ*_n_ (MPa)	Types of Mortar	*f*_c_′ (MPa)	*τ*_e_ (MPa)	*τ*_p_ (MPa)	*τ*_p_/*τ*_e_
Present study	M-0.2	0.2	Mortar	2.1	0.19	0.21	1.12
	M-0.4	0.4			0.26	0.37	1.41
	PM-0.2	0.2	Polymer mortar	34.8	0.27	0.39	1.43
	PM-0.4	0.4			0.53	0.54	1.02
	ECC-0.2	0.2	ECC	22.8	0.32	0.37	1.01
	ECC-0.4	0.4			0.53	0.54	1.00
	CM0.3-0.2	0.2	ECC and mortar (0.3:0.7)	22.8; 2.1	0.28	0.27	1.11
	CM0.3-0.4	0.4			0.42	0.45	1.00
	CM0.5-0.2	0.2	ECC and mortar (0.5:0.5)	22.8; 2.1	0.24	0.23	0.97
	CM0.5-0.4	0.4			0.45	0.39	0.88
Wang’s study [18]	M9-0.2	0.2	Mortar	9.28	0.27	0.27	1.02
	M9-0.4	0.4			0.44	0.43	0.98
	M9-0.6	0.6			0.61	0.59	0.96
	M4-0.2	0.2		3.58	0.24	0.23	0.96
	M4-0.4	0.4			0.40	0.38	0.96
	M4-0.6	0.6			0.54	0.54	1.00
	M0-0.2	0.2		0.39	0.22	0.18	0.82
	M0-0.4	0.4			0.37	0.34	0.91
	M0-0.6	0.6			0.49	0.49	1.00

**Table 6:** Experimental and predicted results of residual shear strength.

Research	Specimen	*σ*_n_ (MPa)	Types of Mortar	*f*_c_′ (MPa)	*τ*_e,r_ (MPa)	*τ*_p,r_ (MPa)	*τ*_p,r0_ (MPa)	*τ*_p,r_/*τ*_e,r_	*τ*_p,r0_/*τ*_e,r_
Present study	M-0.2	0.2	Mortar	2.1	0.13	0.16	0.12	1.17	0.87
	M-0.4	0.4			0.23	0.31	0.23	1.33	0.99
	PM-0.2	0.2	Polymer mortar	34.8	0.17	0.16	0.12	0.93	0.69
	PM-0.4	0.4			0.31	0.31	0.23	1.00	0.74
	ECC-0.2	0.2	ECC	22.8	0.20	0.21	0.16	1.08	0.82
	ECC-0.4	0.4			0.32	0.42	0.32	1.29	0.98
	CM0.3-0.2	0.2	ECC and mortar (0.3:0.7)	22.8; 2.1	0.14	0.17	0.13	1.19	0.89
	CM0.3-0.4	0.4			0.29	0.34	0.26	1.17	0.88
	CM0.5-0.2	0.2	ECC and mortar (0.5:0.5)	22.8; 2.1	0.15	0.18	0.14	1.23	0.92
	CM0.5-0.4	0.4			0.29	0.37	0.27	1.27	0.95
Wang’s study [18]	M9-0.2	0.2	Mortar	9.28	0.18	0.16	0.12	0.86	0.64
	M9-0.4	0.4			0.28	0.31	0.23	1.11	0.83
	M9-0.6	0.6			0.44	0.47	0.35	1.06	0.79
	M4-0.2	0.2		3.58	0.19	0.16	0.12	0.82	0.61
	M4-0.4	0.4			0.27	0.31	0.23	1.15	0.86
	M4-0.6	0.6			0.43	0.47	0.35	1.09	0.81
	M0-0.2	0.2		0.39	0.17	0.16	0.12	0.92	0.68
	M0-0.4	0.4			0.26	0.31	0.23	1.20	0.89
	M0-0.6	0.6			0.40	0.47	0.35	1.17	0.87

## Data Availability

Not applicable.
